# Assessing the added predictive ability of a metabolic syndrome severity score in predicting incident cardiovascular disease and type 2 diabetes: the Atherosclerosis Risk in Communities Study and Jackson Heart Study

**DOI:** 10.1186/s13098-018-0344-3

**Published:** 2018-05-16

**Authors:** Yi Guo, Solomon K. Musani, Mario Sims, Thomas A. Pearson, Mark D. DeBoer, Matthew J. Gurka

**Affiliations:** 1 Department of Health Outcomes and Biomedical Informatics, College of Medicine, University of Florida, Gainesville, FL 32608 USA; 2 Department of Medicine, Jackson Heart Study, University of Mississippi Medical Center, Jackson, MS 39213 USA; 3 Department of Epidemiology, College of Public Health and Health Professions, University of Florida, Gainesville, FL 32608 USA; 4 Division of Pediatric Endocrinology, Department of Pediatrics, University of Virginia, Charlottesville, VA 22908 USA; 5 2004 Mowry Rd Room 3211, PO Box 100177, Gainesville, FL 32610-0177 USA

**Keywords:** Metabolic syndrome, Cardiovascular disease, Type 2 diabetes mellitus, Risk prediction

## Abstract

**Background:**

The severity of the metabolic syndrome (MetS) predicts future coronary heart disease (CHD) and diabetes independent of the individual MetS components. Our aim was to evaluate whether MetS severity conferred additional discrimination to existing scoring systems for cardiovascular disease (CVD) and diabetes risk.

**Methods:**

We assessed Cox proportional hazard models of CHD- and diabetes risk among 13,141 participants of the Atherosclerosis Risk in Communities Study and the Jackson Heart Study, using the Framingham Risk Calculator, the American Heart Association’s Atherosclerotic CVD calculator, the American Diabetes Association diabetes risk score and an additional diabetes risk score derived from ARIC data. We then added a MetS-severity Z-score to these models and assessed for added risk discrimination by assessing Akaike information criterion, c-statistic, integrated discrimination improvement (IDI) and continuous net reclassification improvement (NRI).

**Results:**

The MetS severity score appears to add to the predictive ability of individual CHD and diabetes risk scores. Using the IDI, MetS improved risk prediction for diabetes but not CHD risk. In all 4 scoring systems, MetS severity had a significant non-event NRI, improving the ability to exclude individuals without events. Assessing interactions between risk scores and MetS severity revealed that MetS severity was more highly associated with disease risk among those in the lowest quintiles of risk score, suggesting that MetS was particularly able to identify risk among individuals judged to be of low risk by existing algorithms.

**Conclusions:**

Mets severity improved prediction of diabetes more so than CHD. Incorporation of multiple risk predictors into electronic health records may help in better identifying those at high disease risk, who can then be placed earlier on preventative therapy.

**Electronic supplementary material:**

The online version of this article (10.1186/s13098-018-0344-3) contains supplementary material, which is available to authorized users.

## Background

The continued need for tools to identify risk for future cardiovascular disease (CVD) and Type 2 diabetes mellitus (T2DM) has driven the design of risk-prediction scoring systems, which can then be used to identify and motivate high-risk patients toward preventative treatments and lifestyle improvement. These predictive scoring systems include the Framingham calculator from D’Agostino et al. [1, 2] and the more recent atherosclerotic cardiovascular disease (ASCVD) Pooled Cohort Equations scoring system by Goff et al. [3] (Table 1). These systems can be used clinically to detect future CVD risk, with respectable c statistics of 0.76–0.79 for the Framingham calculator [1] and 0.71–0.82 for the ASCVD [3].

**Table 1:** Predictors included in existing CVD and T2DM risk scores

Predictors	CVD		T2DM	
	Framingham (D’Agostino et al.)	ASCVD (Goff et al.)	Bang et al.	Schmidt et al.
Age	X	X	X	X
Sex	X	X	X	
Race/ethnicity				X
Weight/height			X	X
Waist circumference				X
HDL	X	X		
Total cholesterol	X	X		
Hypertension			X	X
SBP, treated	X	X		
SBP, untreated	X	X		X
Smoking	X	X		
Diabetes	X	X		
Gest. diabetes			X	
Family history of diabetes			X	X
Physical activity			X	
Fasting glucose				X

Scoring systems for risk of future T2DM include that of Schmidt et al. [4], based on prospective data from the Atherosclerosis Risk in Communities Study (ARIC). A predictive score for risk of current T2DM by Bang et al. [5] has been endorsed by the American Diabetes Association (ADA) [6], potentially because of its reliance on a relatively small number of variables and no need for blood testing (Table 1). These T2DM scoring systems detect T2DM risk with an area-under-the-curve of 0.80 for the Schmidt equation [4] 0.74 for the Bang equation [6].

Another notable cardiovascular and diabetes risk predictor is the metabolic syndrome (MetS), a cluster of CVD risk factors including central obesity, high blood pressure, high fasting triglycerides, low HDL cholesterol fasting glucose [7]. These factors are associated with insulin resistance and appear to be driven by underlying processes of adipocyte dysfunction, systemic inflammation and oxidative stress [8]. Using traditional criteria, MetS was classified according to the presence of at least three abnormalities in the MetS components [7]. The value of MetS as a concept had been questioned, with several studies demonstrating that the presence of MetS by traditional criteria did not provide additional CVD or T2DM prediction beyond that conveyed by the individual MetS components [9–11]. We recently formulated a sex- and race/ethnicity-specific MetS severity Z-score with differential weighting of the individual MetS components based on how these components correlated together by sex- and racial/ethnic sub-group [12, 13]. While this MetS severity score was not specifically formulated to be a risk predictor, we demonstrated that this score remained significantly associated with long-term risk for coronary heart disease (CHD) [14, 15] and T2DM [16, 17], even in models that included the individual MetS components [15, 17]—contributing to the notion that the presence of the underlying processes driving MetS may confer additional risk for CHD and T2DM.

Current risk scores do not incorporate a component estimating the presence of MetS, beyond incorporation of some of its individual risk factors. We hypothesized that adding MetS severity to common risk scores for CVD and T2DM would increase their power to predict risk. The goal of this study was to assess (1) whether MetS severity remained a significant predictor of long-term CHD and T2DM risk, even when assessed alongside existing risk scores and (2) whether the addition of MetS to these scores improved their ability to predict CHD risk. We utilized longitudinal data from black and white participants of the Atherosclerosis Risk in Communities (ARIC) study and the Jackson Heart Study (JHS) with up to 20-year follow-up to assess for optimizing of risk via use of MetS severity.

## Methods

### Study sample

The study sample consisted of participants from two large cohort studies: ARIC and the Jackson Heart Study (JHS). This study and/or its analysis was approved by the Institutional Review Boards of the University of Florida, the University of Virginia, and the study sites for the ARIC; all participants provided informed consent. ARIC started in 1987 as a large community-based epidemiological cohort study of mostly white and African–American participants. A total of 15,792 individuals aged 45–64 years old were recruited for four visits across four US sites. JHS started in 2000 as an expansion of the ARIC study site in Jackson, MS, with three visits, focusing on African Americans. JHS recruited 5301 African Americans aged 21–95 years old, among which 1626 participants had been followed as part of ARIC. For these 1626 participants, we used data from their ARIC follow-up and not their JHS follow-up. For the purposes of this analysis, we excluded the participants who self-identified as a race other than white or African American (n = 46). We also excluded participants with baseline (Visit 1) T2DM (n = 2485), CHD (n = 973), or stroke (n = 393), and participants who had missing baseline data on MetS components (n = 792), who had non-fasting laboratory studies (n = 507), and/or those without follow-up data regarding T2DM outcomes (n = 2992). Eventually, data from 13,141 participants were used for this study. Previous reports have published details of procedures for blood collection and analysis for lipids [18] and serum glucose [19]. Briefly, participants fasted overnight for 12 h before the examination. Phlebotomy was performed, blood sample was centrifuged and serum was sent to a central laboratory for examination. Triglycerides were measured by enzymatic methods, and HDL cholesterol was measured after dextran-magnesium precipitation. LDL cholesterol was calculated using the Friedewald equation. Serum glucose was measured by the hexokinase-6 phosphate dehydrogenase method [9]. BP was examined in sitting position with a random-zero sphygmomanometer—of the three measurements performed, the average of the last two measurements were used for analysis.

### Study outcomes

#### Time to incident CHD

Incident CHD was determined from adjudicated outcomes using standard ARIC and JHS protocols and included fatal or nonfatal hospitalized myocardial infarction, fatal CHD, silent myocardial infarction identified by electrocardiography, or coronary revascularization [19, 20]. The study outcome time to incident CHD events was defined as the minimum number of days between the baseline visit and either the first event, death from other causes, last contact, or Dec 31, 2011. Given the focus on evaluating the performance of risk scores that were specifically designed to estimate 10-year risk of CHD, we calculated prediction statistics (described below) of these overall survival models with respect to 10-year risk.

#### T2DM

Incident T2DM was determined slightly differently for the ARIC and JHS participants due to differences in variable specifications. In ARIC, participants were defined as having T2DM if they reported that a physician had told them they had diabetes, had a fasting glucose ≥ 126 mg/dL or a non-fasting glucose ≥ 200 mg/dL, or if they reported they were taking insulin or oral hypoglycemic medications [4]. In JHS, participants were defined as having T2DM if they had a fasting glucose ≥ 126 mg/dL or an HbA1c ≥ 6.5% or if they took a diabetic medication within 2 weeks prior to the clinic visit [21]. Incident T2DM was determined for Visits 2–4 separately or ARIC participants and for Visits 2–3 separately for JHS participants (as we excluded those with T2DM at Visit 1). The study outcome T2DM was defined as a dichotomized variable, with “Yes” being having T2DM during any Visits and “No” being not having T2DM for all Visits, considering a follow-up time of 10 years.

### Predictors: risk scores

#### Existing CVD and T2DM risk scores

We utilized two existing CVD risk scoring algorithms based on Cox regressions for model fitting (Table 1). Using data from the Framingham Heart Study, D’Agostino et al. derived a sex-specific multivariable risk factor algorithm for assessing 10-year general CVD risk [1, 2]. The 2013 American College of Cardiology/American Heart Association Guideline on the Assessment of Cardiovascular Risk (Goff et al. [3]) subsequently published the ASCVD, a sex- and race-specific 10-year ASCVD risk estimation algorithm derived using extensive data from several large, racially and geographically diverse cohort studies, including the Framingham Heart Study, ARIC, the Cardiovascular Health Study, and the Coronary Artery Risk Development in Young Adults (CARDIA) study.

We also utilized two existing T2DM risk scoring algorithms based on logistic regression (Table 1). Bang et al. published a risk scoring algorithm for undiagnosed diabetes developed using the National Health and Nutrition Examination Survey (NHANES) data [5]. The Bang risk algorithm was later adopted by the ADA as the Type 2 diabetes risk test [6]. The other T2DM risk algorithm was developed using ARIC data by Schmidt et al. [4]. For each of these four existing CVD and T2DM risk scores, we converted the scores in the analytic sample to Z-scores for use in the final predictive models.

#### MetS severity score

We calculated the MetS severity Z-scores at baseline for the study participants using sex- and race/ethnicity-based formulas [12]. The MetS severity score was derived from the five traditional MetS components (WC, triglycerides, HDL-cholesterol, systolic BP, fasting glucose) using a factor analysis approach. Because of differences in traditional MetS criteria by race/ethnicity [22–24], confirmatory factor analysis was performed as previously described [12] to determine the weighted contribution of each component to a latent MetS factor on a sex- and race/ethnicity-specific basis, using the National Health and Nutrition Examination Survey (NHANES) data for adults aged 20–64 years. For each of the six sub-groups based on sex and race/ethnicity (non-Hispanic-white, non-Hispanic-black and Hispanic), factor loadings from the five MetS components were determined and used to generate equations for computing a standardized MetS severity score for each sub-group (http://mets.health-outcomes-policy.ufl.edu/calculator/). The MetS severity score was shown to correlate with other MetS risk markers, such as insulin [25] and adiponectin [25], and is predictive of long-term risk of CVD [14, 15] and T2DM [16, 17]. We recently demonstrated that the MetS severity score was predictive of future CHD and T2DM events above and beyond the individual MetS components alone [15, 17]. Because of the importance of insulin resistance in T2DM and CVD, we additionally assessed the homeostasis model of insulin resistance (HOMA-IR) as a risk predictor. HOMA-IR was calculated as: HOMA-IR = (fasting insulin × fasting glucose)/405, where insulin is measured in mU/L and glucose is in mg/dL.

### Statistical analysis

Using data from the combined ARIC and JHS cohorts, we used Cox proportional hazards models to assess the association of existing CVD risk scores and MetS with the time to incident CVD. We used logistic regression models to assess the association of the existing T2DM risk scores and MetS with the incidence of T2DM. To explore the effect of adding MetS on model performance, we used a series of nested models for both Cox and logistic regressions. The predictors in the nested models were: (1) risk score only (Model A); (2) MetS severity only (Model B); (3) risk score and MetS severity (Model C); and (4) risk score, MetS severity, and risk score by MetS severity interaction (Model D). For both outcomes, we also fitted sex- and race-specific models and reported associated odds ratios or hazard ratios. We controlled for study site in all models (four ARIC sites plus JHS). When presenting hazard ratios (HR’s) and odds ratios (OR’s), we standardized the Framingham, ASCVD, and Schmidt risk scores to facilitate comparability with the HR’s and OR’s associated with the MetS severity Z-score. Given the ordinal nature of the Bang diabetes score, we did not standardize for this comparison. Our primary interest was in the model prediction statistics described below and how these risk scores would perform in clinical settings; for these evaluations we used the risk scores on their original scale. All statistical analyses were performed using SAS version 9.4 (SAS, Cary, North Carolina, USA).

Model performance was evaluated using the following statistics: Akaike information criterion (AIC), c statistic, integrated discrimination improvement (IDI), and continuous net reclassification improvement (NRI). The AIC and c statistic were computed for all models. The IDI and continuous NRI were computed for comparing Model C to Model A, and Model D to Model A. The c statistic, and IDI are measures of discrimination, which is a model’s ability to distinguish between subjects with and without the disease. The c statistic is the estimated area under the Receiver Operating Characteristics (ROC) curve. The IDI equals the difference in discrimination slopes between the model with the additional predictor and the model without [26], or the difference in the proportion of variance explained by the two different models [27]. The continuous NRI is a measure of improvement in reclassification, defined as the sum of two differences in proportions resulting from the addition of a new predictor: (1) proportion of individuals with events who have an increase in predicted risks minus the proportion with a decrease (event NRI), and (2) proportion of individuals without events who have a decrease in predicted risks minus the proportion with an increase (non-event NRI). Extensions of the c statistic, IDI, and NRI in the context of survival data have been previously reported [28–30]. While all available follow-up data for CVD was used in our models, we calculated these statistics to evaluate predictive performance at 10 years. Performance statistics, except for the AIC, were computed using validated SAS macros available from: http://ncook.bwh.harvard.edu/sas-macros.html. Finally, variance inflation factors (VIF’s) were computed to assess the degree of collinearity when including MetS and other predictive scores in the same model, with VIF’s greater than 10 representing severe collinearity.

## Results

### Participant characteristics

We summarized the characteristics of the 13,141 study participants at baseline by sex and race in Table 2. The average age of the participants was 53.0 (SD = 7.1) years old. The incidence of CVD at 10 years was the highest among white men (24.6%), compared to 9.4% among white women, 10.3% among black men, and 5.7% among black women. Overall, the incidence of CVD at 10 years was 13.2% for all the participants. The incidence of T2DM at 10 years was 12.0% overall and differed by sex and race. Black men (16.4%) and women (16.5%) had a higher incidence of T2DM, while the rate was 11.7% among white men and 7.9% among white women.

**Table 2:** Characteristics of study participants

Characteristic^a^	Overall	White men	White women	Black men	Black women
n	13,141	3905	4721	1760	2755
Age	53.0 (7.1)	54.5 (5.7)	53.8 (5.6)	51.0 (9.0)	50.8 (8.9)
BMI	27.8 (5.7)	27.2 (3.8)	26.1 (5.0)	28.3 (5.4)	31.4 (7.2)
Waist circumference	95.9 (13.7)	98.9 (10.0)	91.5 (13.6)	97.4 (13.8)	98.1 (16.3)
SBP	120.0 (17.5)	119.2 (15.3)	116.0 (17.2)	126.4 (18.4)	123.6 (18.3)
HDL	52.6 (16.6)	43.6 (12.2)	58.9 (16.8)	48.6 (15.1)	57.1 (16.1)
Triglycerides	118. 5 (73.8)	138.6 (84.6)	119.1 (68.3)	109.5 (77.8)	95.0 (53.1)
Glucose	96.9 (9.7)	100.8 (8.8)	96.6 (8.6)	95.2 (10.3)	93.2 (10.5)
MetS severity score	0.0 (0.8)	0.3 (0.7)	− 0.1 (0.8)	− 0.1 (0.7)	− 0.0 (0.8)
Framingham predicted 10-year CVD risk	0.101 (0.086)	0.150 (0.091)	0.061 (0.049)	0.152 (0.106)	0.069 (0.058)
ASCVD predicted 10-year risk	0.057 (0.053)	0.081 (0.052)	0.029 (0.027)	0.095 (0.062)	0.048 (0.057)
Incident CVD at 10 years; n (%)	1740 (13.2%)	959 (24.6%)	444 (9.4%)	181 (10.3%)	156 (5.7%)
Incident T2DM at 10 years, n (%)	1573 (12.0%)	456 (11.7%)	375 (7.9%)	288 (16.4%)	454 (16.5%)

^a^ All statistics are reported as mean (SD) unless otherwise noted

### CVD risk prediction with CVD risk scores and MetS

Results from adding MetS severity score in addition to the Framingham and ASCVD risk score for predicting future CHD were summarized in Table 3. The CVD risk scores (Model A) and the MetS severity score (Model B) by themselves were significant predictors for future CVD, across the sex and race groups, with an overall HR’s of 2.38 per normalized SD unit increase in Framingham score, 2.68 per normalized SD unit increase in ASCVD and 1.77 per standard deviation unit increase in MetS severity. When included in the same model (Model C), the Framingham score but not MetS severity was a significant predictors for future CHD. Both the ASCVD score and MetS severity were significant predictors for future CHD when included in the same model.

**Table 3:** Cox proportional hazards models: time to incident CVD, overall and by sex and race: risk scores and MetS severity

Model	Framingham risk score (D’Agostino et al. 2008)					ASCVD risk score (Goff et al. 2013)				
	Overall	Sex and race specific analysis				Overall	Sex and race specific analysis			
	n = 12,553	White men n = 3900	White women n = 4715	Black men n = 1478	Black women n = 2460	n = 12,553	White men n = 3900	White women n = 4715	Black men n = 1478	Black women n = 2460
Model A^b^										
CVD risk score HR (95% CI)	*2.38 (2.26, 2.52)*	*2.03 (1.85, 2.22)*	*2.47 (2.20, 2.76)*	*2.49 (2.02, 3.08)*	*2.59 (2.13, 3.15)*	*2.68 (2.51, 2.86)*	*2.27 (2.03, 2.54)*	*2.74 (2.40, 3.12)*	*3.47 (2.56, 4.71)*	*2.43 (2.01, 2.94)*
Model AIC	30,916.55	15,159.02	7166.26	2361.36	2163.07	30,911.43	15,155.87	7146.23	2367.17	2166.56
C statistic	0.72 (0.71, 0.74)	0.64 (0.62, 0.66)	0.70 (0.68, 0.73)	0.71 (0.67, 0.75)	0.72 (0.68, 0.77)	0.72 (0.71, 0.73)	0.64 (0.62, 0.65)	0.71 (0.68, 0.73)	0.70 (0.66, 0.74)	0.73 (0.68, 0.77)
Model B^b^										
MetS severity HR (95% CI)	*1.77 (1.66, 1.88)*	*1.49 (1.37, 1.63)*	*1.86 (1.65, 2.10)*	*1.64 (1.33, 2.02)*	*1.62 (1.32, 2.00)*	*1.77 (1.66, 1.88)*	*1.49 (1.37, 1.63)*	*1.86 (1.65, 2.10)*	*1.64 (1.33, 2.02)*	*1.62 (1.32, 2.00)*
Model AIC	31,394.43	15,265.20	7262.83	2405.99	2224.53	31,394.43	15,265.20	7262.83	2405.99	2224.53
C statistic	0.64 (0.63, 0.65)	0.59 (0.57, 0.60)	0.64 (0.61, 0.66)	0.65 (0.61, 0.69)	0.63 (0.58, 0.67)	0.64 (0.63, 0.65)	0.59 (0.57, 0.60)	0.64 (0.61, 0.66)	0.65 (0.61, 0.69)	0.63 (0.58, 0.67)
Model C^b^										
CVD risk score HR (95% CI)	*2.33 (2.19, 2.48)*	*1.94 (1.74, 2.16)*	*2.41 (2.08, 2.78)*	*2.39 (1.90, 3.01)*	*2.52 (2.04, 3.11)*	*2.54 (2.36, 2.72)*	*2.11 (1.87, 2.39)*	*2.53 (2.17, 2.94)*	*3.14 (2.28, 4.33)*	*2.38 (1.94, 2.91)*
MetS severity HR (95% CI)	1.06 (0.98, 1.14)	1.09 (0.98, 1.21)	1.04 (0.89, 1.22)	1.12 (0.88, 1.42)	1.09 (0.86, 1.38)	*1.14 (1.06, 1.22)*	*1.15 (1.04, 1.27)*	*1.16 (1.00, 1.34)*	*1.29 (1.03, 1.61)*	1.08 (0.85, 1.36)
Model AIC	30,880.50	15,152.18	7159.64	2360.46	2162.74	30,860.53	15,146.65	7136.46	2361.73	2164.39
C statistic	0.72 (0.71, 0.73)	0.64 (0.62, 0.66)	0.70 (0.67, 0.72)	0.71 (0.67, 0.75)	0.72 (0.68, 0.77)	0.72 (0.71, 0.73)	0.64 (0.62, 0.66)	0.70 (0.68, 0.73)	0.70 (0.67, 0.74)	0.72 (0.68, 0.77)
IDI^a^ (95% CI)	− 0.00 (− 0.00, 0.00)	− 0.00 (− 0.00, 0.00)	− 0.00 (− 0.00, 0.00)	− 0.00 (− 0.00, 0.00)	− 0.00 (− 0.00, 0.00)	− 0.00 (− 0.00, 0.00)	0.00 (− 0.00, 0.00)	− 0.00 (− 0.01, 0.00)	− 0.00 (− 0.01, 0.00)	− 0.00 (− 0.01, 0.00)
Continuous NRI^a^ (95% CI)	*0.16 (0.08, 0.23)*	0.02 (− 0.08, 0.13)	*0.22 (0.04, 0.42)*	*0.20 (0.03, 0.45)*	0.19 (− 0.07, 0.42)	*0.15 (0.05, 0.23)*	0.04 (− 0.08, 0.15)	*0.20 (0.01, 0.40)*	*0.24 (0.05, 0.50)*	*0.37 (0.13, 0.62)*
Event NRI^a^ (95% CI)	0.06 (− 0.02, 0.14)	− 0.03 (− 0.14, 0.07)	0.08 (− 0.11, 0.27)	0.14 (− 0.05, 0.36)	0.09 (− 0.17, 0.34)	*0.10 (0.01, 0.18)*	− 0.02 (− 0.12, 0.08)	0.06 (− 0.13, 0.27)	0.16 (− 0.05, 0.40)	*0.30 (0.06, 0.55)*
Non-event NRI^a^ (95% CI)	*0.10 (0.08, 0.11)*	*0.05 (0.03, 0.09)*	*0.14 (0.11, 0.16)*	*0.06 (0.02, 0.11)*	*0.10 (0.05, 0.13)*	*0.05 (0.03, 0.07)*	*0.06 (0.03, 0.08)*	*0.14 (0.11, 0.16)*	*0.08 (0.03, 0.14)*	*0.07 (0.02, 0.10)*
Model D^b^										
CVD × MetS p value	p < 0.0001	p < 0.0001	p < 0.0001	p = 0.4329	p = 0.0095	p < 0.0001	p < 0.0001	p = 0.0032	p = 0.5687	p = 0.1049
Model AIC	30,810.04	15,134.30	7142.84	2361.83	2157.61	30,809.03	15,130.44	7129.02	2363.41	2163.73
C statistic	0.72 (0.71, 0.73)	0.64 (0.62, 0.66)	0.70 (0.67, 0.72)	0.71 (0.67, 0.75)	0.72 (0.68, 0.77)	0.72 (0.71, 0.73)	0.64 (0.62, 0.66)	0.70 (0.68, 0.72)	0.70 (0.66, 0.74)	0.72 (0.68, 0.76)
IDI^a^ (95% CI)	− 0.00 (− 0.01, 0.00)	− 0.00 (− 0.00, 0.00)	− 0.00 (− 0.02, 0.01)	− 0.00 (− 0.00, 0.00)	− 0.00 (− 0.02, 0.01)	− 0.00 (− 0.01, 0.00)	0.00 (− 0.00, 0.00)	− 0.00 (− 0.01, 0.00)	− 0.00 (− 0.01, − 0.00)	− 0.00 (− 0.01, 0.01)
Continuous NRI^a^ (95% CI)	*0.42 (0.35, 0.49)*	*0.20 (0.10, 0.32)*	*0.56 (0.36, 0.73)*	0.14 (− 0.09, 0.48)	*0.48 (0.25, 0.74)*	*0.38 (0.30, 0.45)*	*0.22 (0.10, 0.32)*	*0.46 (0.26, 0.67)*	0.18 (− 0.02, 0.43)	*0.32 (0.03, 0.59)*
Event NRI^a^ (95% CI)	*0.39 (0.32, 0.45)*	*0.33 (0.25, 0.43)*	*0.50 (0.34, 0.67)*	0.09 (− 0.13, 0.39)	*0.46 (0.21, 0.70)*	*0.27 (0.19, 0.34)*	*0.31 (0.23, 0.41)*	*0.38 (0.19, 0.57)*	0.08 (− 0.13, 0.33)	0.17 (− 0.11, 0.43)
Non-event NRI^a^ (95% CI)	*0.03 (0.01, 0.05)*	*− 0.13 (− 0.16, − 0.09)*	*0.06 (0.02, 0.08)*	0.05 (− 0.00, 0.10)	0.02 (− 0.03, 0.06)	*0.11 (0.09, 0.13)*	*− 0.09 (− 0.13, − 0.07)*	*0.08 (0.05, 0.11)*	*0.10 (0.05, 0.15)*	*0.15 (0.11, 0.19)*

All models controlled for study site. The risk scores were standardized to facilitate comparability of HR’s with MetS severity; model fit/prediction statistics included scores on their original scale

Statistically significant (p < 0.05) HR’s (different than 1) and IDI’s/NRI’s (different than 0) were italic for ease of display

^a^ IDI and NRI computed relative to Model A

^b^ Predictors included in models are as follows: Model A: risk score only; Model B: MetS severity only; Model C: risk score and MetS severity; Model D risk score, MetS severity, and risk score by MetS severity interaction

Regarding model performance, there appeared to be mixed indicators of added distinguishing ability when adding MetS severity score to Framingham. In moving from Model A to Model C, there was no change in the c statistic, suggesting the distinguishing ability remaining unchanged with a non-significant IDI. Conversely, the continuous NRI was 0.16 (95% CI 0.08, 0.23), indicating a significant increase in the model’s ability to correctly classify individuals without CVD events (non-event NRI = 0.10; 95% CI 0.08, 0.11) when adding MetS severity. Similar statistics were observed comparing Model D (that included the interaction between the risk score and MetS) to Model A. No major differences in patterns of discrimination and reclassification performance were observed between sex and race groups.

We observed similar results when adding MetS severity score to the ASCVD prediction model. There was no change in c statistic from Model A to Models C and D. Comparing Model C to Model A, the IDI was not significant, while the continuous NRI was 0.15 (95% CI 0.05, 0.23), suggesting a significant increase in reclassification performance. Comparing Model D to Model A, the IDI was not significant, while the continuous NRI was 0.38 (95% CI 0.30, 0.45), again indicating an increase in reclassification performance. Among the sex and race groups, we observed no major differences in patterns of discrimination and reclassification performance. Again, the continuous NRI revealed that adding MetS severity improved the ability to correctly classify individuals without CHD events.

### T2DM risk prediction with T2DM risk scores and MetS

Results from adding MetS severity score in addition to the Bang and Schmidt risk score for predicting incident T2DM were summarized in Table 4. The T2DM risk scores (Model A) and MetS severity score (Model B) were significant predictors for T2DM by themselves, across the sex and race groups. MetS severity was a stronger predictor than the Bang score. When included in the same model (Model C), both the Bang score and MetS severity were significant predictors for T2DM, except in models for women where the Bang score was not significant. In the overall combined model, the Schmidt risk score but not MetS severity was a significant predictors for future T2DM when included in the same model, except in the models for black men and women, in which MetS was more strongly associated and in the model for white men where the MetS severity score was protective.

**Table 4:** Logistic models for predicting type 2 diabetes, overall and by sex and race: risk scores and MetS severity

Model	Bang et al. (2009) risk score					Schmidt et al. (2005) risk score				
	Overall	Sex and race specific analysis				Overall	Sex and race specific analysis			
	n = 13,136	White men n = 3904	White women n = 4721	Black men n = 1758	Black women n = 2753	n = 13,140	White men n = 3904	White women n = 4721	Black men n = 1760	Black women n = 2755
Model A^b^										
T2D risk score OR (95% CI)	*1.99 (1.87, 2.12)*	*2.06 (1.81, 2.34)*	*2.32 (2.04, 2.65)*	*1.86 (1.61, 2.16)*	*1.76 (1.57, 1.97)*	*1.27 (1.25, 1.29)*	*1.32 (1.28, 1.35)*	*1.31 (1.28, 1.34)*	*1.20 (1.17, 1.24)*	*1.24 (1.21, 1.27)*
Model AIC	9007.94	2691.06	2431.27	1501.31	2366.87	7613.79	244.21	1956.73	1360.75	2028.55
C statistic	0.69 (0.68, 0.71)	0.66 (0.64, 0.69)	0.71 (0.69, 0.74)	0.66 (0.63, 0.70)	0.65 (0.63, 0.68)	0.83 (0.82, 0.84)	0.83 (0.81, 0.85)	0.86 (0.84, 0.88)	0.77 (0.74, 0.80)	0.80 (0.78, 0.82)
Model B^b^										
MetS severity OR (95% CI)	*4.01 (3.69, 4.37)*	*2.92 (2.51, 3.41)*	*5.55 (4.68, 6.58)*	*3.86 (3.10, 4.80)*	*4.26 (3.60, 5.03)*	*4.00 (3.67, 4.35)*	*2.92 (2.51, 3.41)*	*5.55 (4.68, 6.58)*	*3.79 (3.05, 4.71)*	*4.24 (3.59, 5.01)*
Model AIC	8252.17	2610.97	2100.56	1396.40	2105.66	8267.56	2610.95	2100.56	1406.21	2110.63
C statistic	0.78 (0.77, 0.79)	0.71 (0.68, 0.74)	0.82 (0.80, 0.84)	0.75 (0.72, 0.78)	0.78 (0.76, 0.80)	0.78 (0.77, 0.79)	0.71 (0.68, 0.74)	0.82 (0.80, 0.84)	0.75 (0.72, 0.78)	0.78 (0.76, 0.80)
Model C^b^										
T2D risk score OR (95% CI)	*1.26 (1.17, 1.35)*	*1.63 (1.42, 1.86)*	1.06 (0.90, 1.25)	*1.34 (1.13, 1.57)*	0.95 (0.82, 1.09)	*1.26 (1.24, 1.29)*	*1.34 (1.30, 1.39)*	*1.29 (1.24, 1.34)*	*1.17 (1.12, 1.21)*	*1.20 (1.16, 1.25)*
MetS severity OR (95% CI)	*3.53 (3.22, 3.88)*	*2.47 (2.10, 2.91)*	*5.35 (4.39, 6.51)*	*3.31 (2.62, 4.18)*	*4.43 (3.64, 5.39)*	1.06 (0.93, 1.20)	0.80 (0.65, 0.97)	1.16 (0.87, 1.55)	*1.41 (1.02, 1.94)*	*1.36 (1.04, 1.78)*
Model AIC	8216.11	2563.08	2102.05	1386.36	2107.08	7593.16	2245.49	1949.91	1351.12	2010.50
C statistic	0.78 (0.77, 0.79)	0.73 (0.71, 0.75)	0.82 (0.80, 0.84)	0.75 0.72, 0.78)	0.78 (0.76, 0.80)	0.83 (0.82, 0.84)	0.83 (0.81, 0.85)	0.86 (0.84, 0.88)	0.78 (0.75, 0.81)	0.81 (0.79, 0.83)
IDI^a^ (95% CI)	*0.07 (0.06, 0.08)*	*0.04 (0.03, 0.05)*	*0.11 (0.09, 0.12)*	*0.07 (0.06, 0.09)*	*0.10 (0.09, 0.11)*	*− 0.00 (− 0.00, 0.00)*	0.00 (0.00, 0.00)	− 0.00 (− 0.01, 0.00)	*0.00 (− 0.00, 0.01)*	0.00 (− 0.00, 0.00)
Continuous NRI^a^ (95% CI)	*0.68 (0.63, 0.72)*	*0.55 (0.44, 0.66)*	*0.82 (0.70, 0.91)*	*0.60 (0.47, 0.70)*	*0.75 (0.67, 0.87)*	*0.16 (0.12, 0.22)*	*− 0.02 (− 0.14, 0.07)*	*0.20 (0.09, 0.32)*	*0.20 (0.09, 0.31)*	*0.22 (0.11, 0.32)*
Event NRI^a^ (95% CI)	*0.34 (0.30, 0.38)*	*0.32 (0.23, 0.43)*	*0.40 (0.29, 0.48)*	*0.30 (0.18, 0.38)*	*0.39 (0.31, 0.49)*	0.02 (− 0.02, 0.07)	*− 0.02 (− 0.11, 0.06)*	− 0.01 (− 0.12, 0.11)	*0.11 (0.02, 0.22)*	0.04 (− 0.05, 0.14)
Non-event NRI^a^ (95% CI)	*0.34 (0.32, 0.36)*	*0.23 (0.19, 0.26)*	*0.42 (0.39, 0.45)*	*0.30 (0.26, 0.36)*	*0.36 (0.32, 0.40)*	*0.14 (0.12, 0.16)*	− 0.00 (− 0.04, 0.04)	*0.21 (0.18, 0.24)*	*0.09 0.03, 0.13)*	*0.18 (0.13, 0.22)*
Model D^b^										
T2D × MetS p value	p = 0.0002	p = 0.0339	p = 0.1879	p = 0.8658	p = 0.0025	p < 0.0001	p < 0.0001	p < 0.0001	p < 0.0001	p < 0.0001
Model AIC	8203.45	2560.50	2102.27	1388.34	2099.40	7480.67	2210.59	1924.08	1337.13	1986.52
C statistic	0.78 (0.77, 0.79)	0.73 (0.71, 0.76)	0.82 (0.80, 0.84)	0.75 (0.72, 0.78)	0.78 (0.76, 0.80)	0.83 (0.82, 0.84)	0.83 (0.81, 0.85)	0.86 (0.84, 0.88)	0.78 (0.75, 0.81)	0.81 (0.79, 0.83)
IDI^a^ (95% CI)	*0.07 (0.06, 0.08)*	*0.04 (0.03, 0.05)*	*0.10 (0.09, 0.12)*	*0.07 (0.06, 0.09)*	*0.10 (0.09, 0.12)*	0.00 (− 0.00, 0.01)	0.01 (− 0.00, 0.01)	− 0.00 (− 0.01, 0.00)	*0.01 (0.00, 0.02)*	0.00 (− 0.00, 0.01)
Continuous NRI^a^ (95% CI)	*0.68 (0.64, 0.74)*	*0.58 (0.48, 0.69)*	*0.83 (0.71, 0.92)*	*0.59 (0.43, 0.69)*	*0.75 (0.66, 0.87)*	*0.47 (0.41, 0.52)*	*0.48 (0.39, 0.58)*	*0.56 (0.47, 0.69)*	*0.48 (0.35, 0.59)*	*0.41 (0.30, 0.48)*
Event NRI^a^ (95% CI)	*0.32 (0.29, 0.37)*	*0.32 (0.23, 0.42)*	*0.40 (0.29, 0.50)*	*0.29 (0.15, 0.37)*	*0.37 (0.28, 0.47)*	*0.31 (0.26, 0.36)*	*0.33 (0.23, 0.42)*	*0.28 (0.19, 0.40)*	*0.37 (0.26, 0.44)*	*0.25 (0.15, 0.34)*
Non-event NRI^a^ (95% CI)	*0.36 (0.34, 0.38)*	*0.26 (0.22, 0.29)*	*0.43 (0.41, 0.46)*	*0.30 (0.25, 0.35)*	*0.38 (0.34, 0.42)*	*0.16 (0.14, 0.17)*	*0.15 (0.11, 0.19)*	*0.28 (0.25, 0.31)*	*0.11 (0.06, 0.17)*	*0.16 (0.12, 0.20)*

All models controlled for study site. The risk scores were standardized to facilitate comparability of OR’s with MetS severity; model fit/prediction statistics included scores on their original scale

Statistically significant (p < 0.05) OR’s (different than 1) and IDI’s/NRI’s (different than 0) were italic for ease of display

^a^ IDI and NRI computed relative to Model A

^b^ Predictors included in models are as follows: Model A: risk score only; Model B: MetS severity only; Model C: risk score and MetS severity; Model D risk score, MetS severity, and risk score by MetS severity interaction

When adding MetS severity score as a new predictor in addition to the Bang score (from Model A to Model C), there was a significant increase in the c statistic, suggesting added discrimination. The c statistic for Models A and C were 0.69 (95% CI 0.68, 0.71) and 0.78 (95% CI 0.77, 0.79), respectively. Similarly, the comparison of Model C with Model A, for which the IDI was 0.07 (95% CI 0.06, 0.08), suggests a large increase in MetS severity’s ability to distinguish participants with and without T2DM relative to the Bang score. The continuous NRI was 0.68 (95% CI 0.63, 0.72), suggesting a significant increase in model’s ability to correctly classify individuals with or without T2DM. The performance of Model D was similar to that of Model C.

We observed smaller increases in discrimination and reclassification performance when adding MetS severity score to the Schmidt score for predicting incident T2DM (Table 4). Comparing Model C to Model A, the c statistic remained unchanged, and the IDI was statistically non-significant (− 0.00; 95% CI − 0.00, 0.00). However, the continuous NRI was 0.16 (95% CI 0.12, 0.22), indicating a significant increase in the model’s ability to correctly classify individuals with or without T2DM when adding MetS severity. Model D had a better performance than Model C, with the continuous NRI being 0.47 (95% CI 0.41, 0.52). Across all analyses of T2DM models (Table 4), there were no major differences in model performance among the sex and race groups.

### HOMA-IR and risk prediction

As a risk predictor, HOMA-IR was not as strongly linked to future CVD or T2DM as was MetS in individual models (Additional file 1: Tables S1 and S2). In the combined models, HOMA-IR remained linked to future CVD to a similar extent as seen for MetS-Z. For T2DM models, HOMA-IR remained linked to future T2DM when assessed alongside both diabetes scores.

### Interactions between Risk Scores and MetS

**Fig. 1:**
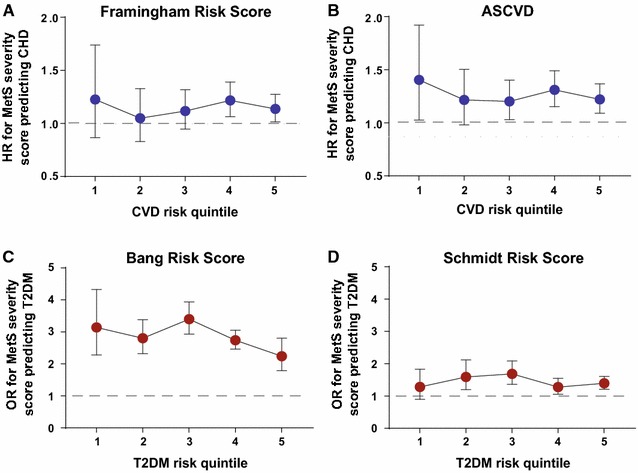
Interaction between risk scores and MetS severity score by disease risk score quintiles. Hazard ratios (HR) or odds ratios (OR) for each MetS severity score Z-score standard deviation unit by quintiles of risk score for cardiovascular disease (CVD) (**a** Framingham risk score; **b** ASCVD score) and Type 2 diabetes mellitus (T2DM) (**c** Bang score (Ref. [5]); **d** Schmidt score (Ref. [4])). For each of the comparator risk scores, there was a significant interaction between MetS severity and future disease risk depending on the quintile of the comparator score, with increasing MetS severity exhibiting higher hazard ratios (HR) for future CHD among individuals in the lowest quintile of CVD risk according to the Framingham and ASCVD risk and with increasing MetS severity exhibiting higher HR’s for future diabetes among individuals in the middle quintiles of diabetes risk according to the Bang and Schmidt risk scores. All models controlled for study site

In the overall and sex/race specific models for predicting CHD and T2DM, we also fitted the interaction term (i.e., how the relationship between MetS severity a future disease diagnosis varied by the level of the comparator risk score) between the respective disease risk score (by quintiles) and MetS severity score (Model D). In each of the four overall prediction models (2 for CVD and 2 for T2DM), the risk score by MetS interaction was statistically significant, demonstrating that MetS severity performed differently in risk assessment depending on the underlying score. We summarized the interaction plots from the four models in Fig. 1. As seen in Fig. 1a, b, the hazard ratios of MetS assumed an s-shape across the CVD risk quintiles based on either the Framingham or ASCVD score. MetS was a stronger predictor for CHD among individuals with the lowest CVD risk (quintile 1). Then, it became a relatively weaker predictor for CHD among individuals with higher CVD risk (quintiles 2 and 3), before becoming a stronger predictor among individuals with higher CVD risk in quintile 4. As seen in Fig. 1c, d, the odds ratios of MetS differed across the T2DM risk quintiles. In the model with the Bang score, MetS was a stronger predictor for T2DM for the middle risk quintile, but a weaker predictor among individuals with the highest T2DM risk. In the model with the Schmidt score, MetS was a stronger predictor for T2DM for the middle risk quintile, but a weaker predictor among individuals with higher T2DM risk (quintiles 4 and 5).

## Discussion

We found that a MetS severity Z-score, when added to predictive models alongside existing T2DM risk scores, consistently improved the models’ discrimination performance for future T2DM. This contrasted with the MetS severity when applied to CHD events: while the MetS severity score remained significantly associated with future CHD when added to models with the ASCVD risk score, this addition did not result in a consistent improvement in the prediction models’ discrimination performance and overall performed best at reclassifying individuals without CHD events. We had previously demonstrated that a MetS severity Z-score was associated with CHD and T2DM outcomes, even in models that included the individual MetS components [15, 17]; however, when tested in models without the individual components, this association was much stronger when comparing the 4th vs. the 1st quartile of MetS severity for future T2DM (HR = 17.4 over 8 years) than with CHD (HR = 4.0 over 25 years follow-up) [15, 17]. The current results reveal that the same hierarchy exists when adding MetS severity for existing scores—that the utility in adding the MetS severity score to improve clinical accuracy is much clearer for T2DM than for CVD.

It is important to note that the existing CVD- and T2DM risk scoring systems that we assessed in the current study were formulated specifically to predict long-term CVD events or diabetes diagnosis using known risk factors (but without incorporating an estimate of MetS beyond its individual components) [1, 3–5]. By contrast, the MetS severity Z-score was formulated not based on CVD or T2DM risk *per se* but on how the individual MetS variables cluster together—potentially as an estimate of the pathological processes underlying MetS, such as adipocyte cellular dysfunction, systemic inflammation, and oxidative stress [8]. Nevertheless, adding the MetS severity score to existing T2DM risk scores in incident diabetes models revealed that this estimate of MetS severity increased the discrimination performance of models, especially for the score from Bang et al. In the prediction models for both CVD and T2DM outcomes, adding MetS severity score to existing risk scores increased the NRI assessment of the models’ ability to correctly reclassify individuals with or without the disease. This suggests that the MetS score has identified risk associated with the way that these individual components are clustered—and that this MetS factor is distinct from risk associated with the existing T2DM scores.

Both of the CVD scoring systems that we evaluated had similar risk prediction for CHD that exceeded that of the MetS severity Z-score. This is not surprising given the inclusion in both of these CVD scores of smoking and LDL cholesterol—clearly important non-MetS-related risk factors that themselves carry strong associations with future CHD. Nevertheless, MetS severity predictive ability was strongest among individuals who were in the lowest risk category based on the CVD scoring systems—suggesting a role for MetS severity as a follow-up test among individuals previously identified as low risk by the scoring systems themselves. This was true for the Bang T2DM risk score also as well as for those in the second and third quintiles of the Schmidt score.

Despite the availability of web-based calculators, clinical use of scoring systems such as these remains limited by the time required to calculate the score on a per-patient basis. Thus, addition of an extra layer of complexity of an additional factor such as MetS severity is at first intimidating. Nevertheless, automated calculation of such scores using the electronic health record (EHR) could facilitate wider use toward identification of high-risk patients. In addition to laboratory values, smoking status is widely available as a codified item through meaningful-use programs. Similar risk-identification algorithms are already widely utilized in EHR systems. Our data suggest the potential that MetS severity could be calculated automatically for use in such systems.

MetS has strong associations with insulin resistance, which itself is linked to risk for T2DM [31] and CVD [32]. It is thus perhaps not surprising that HOMA-IR [33] also had associations with these outcomes. Unfortunately, HOMA-IR has had difficulties in clinical application, as it relies on measurement of insulin. Insulin still does not have a standardized laboratory approach, with measured outcome varying significantly between laboratory assays. However, in research studies using a single insulin assessment technique, levels of insulin are associated with these outcomes of interest. None of the risk scores that we assessed here included insulin measure, potentially explaining its persistent association here.

This study had multiple limitations. The cohorts represented here were initially enrolled 12–29 years ago, at a time when many current CVD treatments and management approaches were not available or not in common use. Thus, the precise predictive ability represented here is likely not generalizable to modern populations. In addition, the definition of Type 2 diabetes differed between the two cohorts, with JHS including elevated HbA1c as an indicator of diabetes—potentially identifying more individuals who had previously-unidentified diabetes but may have had normal fasting blood glucose. However, we adjusted all of these analyses for study site; thus, if there were any differences in the relationship between risk score and outcome according to the method of diabetes diagnosis, this would have been accounted. In this analysis we did not examine the relative performance of MetS severity in predicting future disease among the myriad other risk scores available for CVD and T2DM outcomes. To address our primary aims of examining the utility of MetS severity in predicting future disease above and beyond existing risk scores, we selected some of the more prominently studied and used risk score algorithms. Some of these risk equations (the ASCVD and the Schmidt prediction of T2DM) utilized ARIC in their development; thus, comparisons between these and other scores themselves may not be appropriate. However, our primary goal was to evaluate the added value of risk prediction associated with MetS severity, and using popular equations was an initial step in this process. While it is plausible major differences in our conclusions would result if we examined other algorithms, given the similarity in the composition of most of the algorithms, we suspect similar findings regarding our hypotheses regarding MetS severity and prediction of future disease. In addition, there are participants with less than 10 years of follow-up, particularly with respect to reliable T2DM diagnosis, which would impact the predictive ability of the 10-year risk scores. Nevertheless, any associated bias would not differ among the scores themselves, nor would the ability to determine if MetS severity adds any predictive benefit.

## Conclusions

We found that adding MetS severity—potentially as a marker of underlying metabolic disarray—improved the predictive performance of risk scores for future T2DM much more consistently than for CVD. These data are significant in providing the potential to strengthen current scoring systems via the addition of an estimate of MetS severity—potentially adding accuracy to the identification of individuals at high disease risk, who can then be more appropriately targeted for treatment.

## Additional file

**Additional file 1: Table S1.** Cox Proportional Hazards Models: Time to Incident CVD, Overall and by Sex and Race: Risk Scores and HOMA-IR. **Table S2.** Logistic Models for Predicting Type 2 Diabetes, Overall and by Sex and Race: Risk Scores and HOMA-IR.

## Abbreviations

AIC: Akaike information criterion
ARIC: Atherosclerosis Risk in Communities
ASCVD: atherosclerotic cardiovascular disease
ATP-III: Adult Treatment Panel III
BP: blood pressure
CARDIA: Coronary Artery Risk Development in Young Adults
CHD: coronary heart disease
CVD: cardiovascular disease
IDI: integrated discrimination improvement
MetS: metabolic syndrome
NHANES: National Health and Nutrition Examination Survey
NRI: continuous net reclassification improvement
T2DM: Type 2 diabetes mellitus
VIF: variance inflation factor
WC: waist circumference
