# Supplementary material for: Assessing the added predictive ability of a metabolic syndrome severity score in predicting incident cardiovascular disease and type 2 diabetes: the Atherosclerosis Risk in Communities Study and Jackson Heart Study
Source: Diabetol Metab Syndr. 2018 May 16;10:42. doi: 10.1186/s13098-018-0344-3 (PMC5956946; doi:10.1186/s13098-018-0344-3)
Supplement: Supplementary file 1 — Additional file 1: Table S1. Cox Proportional Hazards Models: Time to Incident CVD, Overall and by Sex and Race: Risk Scores and HOMA-IR. Table S2. Logistic Models for Predicting Type 2 Diabetes, Overall and by Sex and Race: Risk Scores and HOMA-IR. [file 13098_2018_344_MOESM1_ESM.docx]

**Table S1. Cox Proportional Hazards Models: Time to Incident CVD, Overall and by Sex and Race: Risk Scores and HOMA-IR**

| Model | Framingham Risk Score (D’Agostino et al., 2008) | | | | | |  | ASCVD Risk Score (Goff et al., 2013) | | | | | |
| --- | --- | --- | --- | --- | --- | --- | --- | --- | --- | --- | --- | --- | --- |
|  | Overall |  | Sex and Race Specific Analysis | | | |  | Overall |  | Sex and Race Specific Analysis | | | |
|  | n=12,553 |  | White Men  n=3,900 | White Women  n=4,715 | Black Men  n=1,478 | Black Women  n=2,460 |  | n=12,553 |  | White Men  n=3,900 | White Women  n=4,715 | Black Men  n=1,478 | Black Women  n=2,460 |
| Model A** |  |  |  |  |  |  |  |  |  |  |  |  |  |
| CVD risk score HR  (95% CI) | 2.38  (2.26, 2.52) |  | 2.03  (1.85, 2.22) | 2.47  (2.20, 2.76) | 2.49  (2.02, 3.08) | 2.59  (2.13, 3.15) |  | 2.68  (2.51, 2.86) |  | 2.27  (2.03, 2.54) | 2.74  (2.40, 3.12) | 3.47  (2.56, 4.71) | 2.43  (2.01, 2.94) |
| Model AIC | 30916.55 |  | 15159.02 | 7166.26 | 2361.36 | 2163.07 |  | 30911.43 |  | 15155.87 | 7146.23 | 2367.17 | 2166.56 |
| C Statistic | 0.72  (0.71, 0.74) |  | 0.64  (0.62, 0.66) | 0.70  (0.68, 0.73) | 0.71  (0.67, 0.75) | 0.72  (0.68, 0.77) |  | 0.72  (0.71, 0.73) |  | 0.64  (0.62, 0.65) | 0.71  (0.68, 0.73) | 0.70  (0.66, 0.74) | 0.73  (0.68, 0.77) |
| Model B** |  |  |  |  |  |  |  |  |  |  |  |  |  |
| Log-HOMA-IR HR  (95% CI) | 1.29  (1.22, 1.35) |  | 1.15  (1.08, 1.23) | 1.34  (1.21, 1.47) | 1.27  (1.10, 1.47) | 1.12  (0.97, 1.29) |  | 1.29  (1.22, 1.35) |  | 1.15  (1.08, 1.23) | 1.34  (1.21, 1.47) | 1.27  (1.10, 1.47) | 1.12  (0.97, 1.29) |
| Model AIC | 31653.65 |  | 15329.05 | 7342.16 | 2414.33 | 2244.65 |  | 31653.65 |  | 15329.05 | 7342.16 | 2414.33 | 2244.65 |
| C Statistic | 0.58  (0.57, 0.60) |  | 0.55  (0.53, 0.57) | 0.58  (0.55, 0.61) | 0.62  (0.58, 0.66) | 0.58  (0.53, 0.63) |  | 0.58  (0.57, 0.60) |  | 0.55  (0.53, 0.57) | 0.58  (0.55, 0.61) | 0.62  (0.58, 0.66) | 0.58  (0.53, 0.63) |
| Model C** |  |  |  |  |  |  |  |  |  |  |  |  |  |
| CVD risk score HR  (95% CI) | 2.36  (2.23, 2.50) |  | 2.01  (1.83, 2.21) | 2.44  (2.16, 2.75) | 2.43  (1.96, 3.01) | 2.59  (2.13, 3.15) |  | 2.63  (2.46, 2.82) |  | 2.24  (2.00, 2.51) | 2.66  (2.32, 3.05) | 3.36  (2.47, 4.57) | 2.43  (2.01, 2.95) |
| Log-HOMA-IR HR  (95% CI) | 1.04  (0.99, 1.09) |  | 1.02  (0.96, 1.09) | 1.03  (0.93, 1.14) | 1.14  (0.99, 1.31) | 1.00  (0.86, 1.16) |  | 1.06  (1.01, 1.12) |  | 1.04  (0.98, 1.11) | 1.08  (0.98, 1.19) | 1.19  (1.03, 1.37) | 0.99  (0.86, 1.15) |
| Model AIC | 30904.78 |  | 15158.58 | 7165.66 | 2356.00 | 2165.01 |  | 30895.83 |  | 15154.82 | 7144.93 | 2359.24 | 2168.55 |
| C Statistic | 0.72  (0.71, 0.74) |  | 0.64  (0.62, 0.66) | 0.70  (0.68, 0.73) | 0.71  (0.67, 0.75) | 0.72  (0.68, 0.77) |  | 0.72  (0.71, 0.73) |  | 0.64  (0.62, 0.66) | 0.71  (0.68, 0.73) | 0.70  (0.66, 0.74) | 0.73  (0.68, 0.77) |
| IDI*  (95% CI) | 0.00  (-0.00, 0.00) |  | 0.00  (-0.00, 0.00) | -0.00  (-0.00, 0.00) | 0.00  (-0.00, 0.01) | 0.00  (-0.00, 0.00) |  | 0.00  (-0.00, 0.00) |  | 0.00  (-0.00, 0.00) | -0.00  (-0.00, 0.00) | 0.00  (0.00, 0.01) | -0.00  (-0.00, 0.00) |
| Continuous NRI*  (95% CI) | 0.16  (0.08, 0.25) |  | 0.07  (-0.02, 0.20) | 0.17  (-0.04, 0.33) | 0.10  (-0.12, 0.37) | -0.23  (-0.49, 0.03) |  | 0.14  (0.06, 0.23) |  | 0.06  (-0.03, 0.19) | 0.15  (-0.02, 0.36) | 0.16  (-0.05, 0.43) | 0.25  (-0.01, 0.51) |
| Event NRI*  (95% CI) | -0.17  (-0.26, -0.09) |  | -0.24  (-0.35, -0.13) | -0.17  (-0.38, -0.01) | -0.28  (-0.47, -0.03) | -0.00  (-0.27, 0.25) |  | -0.16  (-0.24, -0.07) |  | -0.25  (-0.35, -0.14) | -0.15  (-0.32, 0.05) | -0.22  (-0.42, 0.01) | 0.01  (-0.25, 0.28) |
| Non-event NRI*  (95% CI) | 0.33  (0.31, 0.35) |  | 0.32  (0.30, 0.35) | 0.34  (0.32, 0.37) | 0.38  (0.34, 0.43) | -0.23  (-0.27, -0.19) |  | 0.30  (0.28, 0.32) |  | 0.32  (0.29, 0.34) | 0.30  (0.28, 0.33) | 0.38  (0.34, 0.43) | 0.24  (0.21, 0.29) |
| Model D** |  |  |  |  |  |  |  |  |  |  |  |  |  |
| CVD × Log-HOMA p-value | P= 0.6951 |  | P=0.1166 | P=0.4895 | p=0.0149 | p=0. 1978 |  | P=0.9275 |  | P=0.2180 | p=0.0478 | p=0.1087 | p=0.3541 |
| Model AIC | 30901.46 |  | 15156.84 | 7161.08 | 2356.80 | 2165.81 |  | 30888.27 |  | 15152.90 | 7140.27 | 2360.68 | 2170.43 |
| C Statistic | 0.72  (0.71, 0.73) |  | 0.64  (0.62, 0.66) | 0.70  (0.68, 0.72) | 0.71  (0.67, 0.75) | 0.72  (0.68, 0.77) |  | 0.72  (0.71, 0.73) |  | 0.64  (0.62, 0.66) | 0.70  (0.68, 0.73) | 0.70  (0.66, 0.74) | 0.73  (0.68, 0.77) |
| IDI*  (95% CI) | -0.00  (-0.00,- 0.0) |  | -0.00  (-0.00, 0.00) | -0.00  (-0.00, 0.01) | 0.01  (-0.00, 0.02) | -0.00  (-0.01, 0.00) |  | -0.00  (-0.00, 0.00) |  | -0.00  (-0.00, 0.00) | 0.00  (-0.00, 0.01) | 0.01  (-0.00, 0.01) | -0.00  (-0.00, 0.00) |
| Continuous NRI*  (95% CI) | 0.19  (0.11, 0.26) |  | 0.17  (0.08, 0.29) | 0.52  (0.38, 0.73) | 0.04  (-0.19, 0.33) | 0.38  (0.00, 0.61) |  | 0.24  (0.15, 0.33) |  | 0.19  (0.11, 0.29) | 0.54  (0.32, 0.76) | 0.18  (-0.06, 0.44) | 0.30  (0.10, 0.52) |
| Event NRI*  (95% CI) | -0.11  (-0.18, -0.04) |  | -0.04  (-0.14, 0.08) | 0.32  (0.18, 0.51) | -0.25  (-0.45, 0.03) | 0.23  (-0.14, 0.46) |  | -0.06  (-0.15, 0.02) |  | -0.03  (-0.12, -0.07) | 0.29  (0.07, 0.51) | -0.19  (-0.39, 0.04) | 0.05  (-0.15, 0.30) |
| Non-event NRI*  (95% CI) | 0.31  (0.29, 0.32) |  | 0.21  (0.18, 0.23) | 0.20  (0.17, 0.23) | 0.29  (0.23, 0.34) | 0.15  (0.10, 0.19) |  | 0.30  (0.28, 0.32) |  | 0.22  (0.19, 0.24) | 0.25  (0.22, 0.28) | 0.37  (0.33, 0.42) | 0.24  (0.20, 0.28) |

*IDI and NRI computed relative to Model A

** Predictors included in models are as follows: Model A: risk score only; Model B: HOMA-IR only; Model C: risk score and HOMA-IR; Model D risk score, HOMA-IR, and risk score by HOMA-IR interaction.

All models controlled for study site. HOMA-IR was standardized to facilitate comparability of HR’s with MetS severity; model fit/prediction statistics included scores on their original scale.

Statistically significant (p < 0.05) HR’s (different than 1) and IDI’s/NRI’s (different than 0) were shaded for ease of display

**Table S2. Logistic Models for Predicting Type 2 Diabetes, Overall and by Sex and Race: Risk Scores and HOMA-IR**

| Model | Bang et al. 2009 Risk Score | | | | | |  | Schmidt et al. 2005 Risk Score | | | | | |
| --- | --- | --- | --- | --- | --- | --- | --- | --- | --- | --- | --- | --- | --- |
|  | Overall |  | Sex and Race Specific Analysis | | | |  | Overall |  | Sex and Race Specific Analysis | | | |
|  | n=13,136 |  | White Men  n=3,904 | White Women  n=4,721 | Black Men  n=1,758 | Black Women  n=2,753 |  | n=13,140 |  | White Men  n=3,904 | White Women  n=4,721 | Black Men  n=1,760 | Black Women  n=2,755 |
| Model A** |  |  |  |  |  |  |  |  |  |  |  |  |  |
| T2D risk score OR  (95% CI) | 1.99  (1.87, 2.12) |  | 2.06  (1.81, 2.34) | 2.32  (2.04, 2.65) | 1.86  (1.61, 2.16) | 1.76  (1.57, 1.97) |  | 1.27  (1.25, 1.29) |  | 1.32  (1.28, 1.35) | 1.31  (1.28, 1.34) | 1.20  (1.17, 1.24) | 1.24  (1.21, 1.27) |
| Model AIC | 9007.94 |  | 2691.06 | 2431.27 | 1501.31 | 2366.87 |  | 7613.79 |  | 2244.21 | 1956.73 | 1360.75 | 2028.55 |
| C Statistic | 0.69  (0.68, 0.71) |  | 0.66  (0.64, 0.69) | 0.71  (0.69, 0.74) | 0.66  (0.63, 0.70) | 0.65  (0.63, 0.68) |  | 0.83  (0.82, 0.84) |  | 0.83  (0.81, 0.85) | 0.86  (0.84, 0.88) | 0.77  (0.74, 0.80) | 0.80  (0.78, 0.82) |
| Model B** |  |  |  |  |  |  |  |  |  |  |  |  |  |
| Log-HOMA-IR OR  (95% CI) | 4.01  (3.69, 4.37) |  | 2.92  (2.51, 3.41) | 5.55  (4.68, 6.58) | 3.86  (3.10, 4.80) | 4.26  (3.60, 5.03) |  | 2.88  (2.69, 3.08) |  | 2.51  (2.23, 2.81) | 3.46  (3.02, 3.95) | 2.40  (2.05, 2.81) | 2.57  (2.27, 2.91) |
| Model AIC | 8252.17 |  | 2610.97 | 2100.56 | 1396.40 | 2105.66 |  | 8665.04 |  | 2590.32 | 2297.77 | 1464.76 | 2272.94 |
| C Statistic | 0.78  (0.77, 0.79) |  | 0.71  (0.68, 0.74) | 0.82  (0.80, 0.84) | 0.75  (0.72, 0.78) | 0.78  (0.76, 0.80) |  | 0.76  (0.75, 0.77) |  | 0.73  (0.71, 0.76) | 0.79  (0.77, 0.82) | 0.72  (0.69, 0.75) | 0.74  (0.72, 0.77) |
| Model C** |  |  |  |  |  |  |  |  |  |  |  |  |  |
| T2D risk score OR  (95% CI) | 1.26  (1.17, 1.35) |  | 1.63  (1.42, 1.86) | 1.06  (0.90, 1.25) | 1.34  (1.13, 1.57) | 0.95  (0.82, 1.09) |  | 1.23  (1.22, 1.25) |  | 1.29  (1.25, 1.33) | 1.27  (1.23, 1.30) | 1.17  (1.13, 1.20) | 1.20  (1.17, 1.24) |
| Log-HOMA-IR OR  (95% CI) | 3.53  (3.22, 3.88) |  | 2.47  (2.10, 2.91) | 5.35  (4.39, 6.51) | 3.31  (2.62, 4.18) | 4.43  (3.64, 5.39) |  | 1.36  (1.26, 1.48) |  | 1.21  (1.05, 1.39) | 1.38  (1.16, 1.63) | 1.39  (1.16, 1.67) | 1.42  (1.22, 1.64) |
| Model AIC | 8216.11 |  | 2563.08 | 2102.05 | 1386.36 | 2107.08 |  | 7568.18 |  | 2233.12 | 1953.52 | 1350.47 | 2013.76 |
| C Statistic | 0.78  (0.77, 0.79) |  | 0.73  (0.71, 0.75) | 0.82  (0.80, 0.84) | 0.75  (0.72, 0.78) | 0.78  (0.76, 0.80) |  | 0.83  (0.82, 0.84) |  | 0.83  (0.81, 0.85) | 0.86  (0.84, 0.88) | 0.78  (0.75, 0.81) | 0.81  (0.79, 0.83) |
| IDI*  (95% CI) | 0.07  (0.06, 0.08) |  | 0.04  (0.03, 0.05) | 0.11  (0.09, 0.12) | 0.07  (0.06, 0.09) | 0.10  (0.09, 0.11) |  | 0.00  (0.00, 0.01) |  | 0.00  (-0.00, 0.01) | 0.00  (-0.00, 0.00) | 0.01  (0.00, 0.01) | 0.00  (-0.00, 0.01) |
| Continuous NRI*  (95% CI) | 0.68  (0.63, 0.72) |  | 0.55  (0.44, 0.66) | 0.82  (0.70, 0.91) | 0.60  (0.47, 0.70) | 0.75  (0.67, 0.87) |  | 0.28  (0.22, 0.34) |  | 0.22  (0.14, 0.33) | 0.25  (0.12, 0.37) | 0.32  (0.20, 0.46) | 0.33  (0.22, 0.43) |
| Event NRI*  (95% CI) | 0.34  (0.30, 0.38) |  | 0.32  (0.23, 0.43) | 0.40  (0.29, 0.48) | 0.30  (0.18, 0.38) | 0.39  (0.31, 0.49) |  | -0.08  (-0.12, -0.02) |  | -0.15  (-0.23, -0.06) | -0.03  (-0.14, 0.09) | -0.05  (-0.16, 0.08) | -0.06  (-0.16, 0.04) |
| Non-event NRI*  (95% CI) | 0.34  (0.32, 0.36) |  | 0.23  (0.19, 0.26) | 0.42  (0.39, 0.45) | 0.30  (0.26, 0.36) | 0.36  (0.32, 0.40) |  | 0.36  (0.34, 0.37) |  | 0.37  (0.33, 0.40) | 0.28  (0.25, 0.30) | 0.37  (0.31, 0.42) | 0.39  (0.35, 0.42) |
| Model D** |  |  |  |  |  |  |  |  |  |  |  |  |  |
| T2D × Log-HOMA p-value | p=0.0002 |  | p=0.0339 | p=0.1879 | p=0.8658 | p=0.0025 |  | P=0.4967 |  | P=0.9711 | P=0.1588 | P=0.0412 | P=0.6103 |
| Model AIC | 8203.45 |  | 2560.50 | 2102.27 | 1388.34 | 2099.40 |  | 7475.54 |  | 2211.05 | 1917.55 | 1347.22 | 1993.40 |
| C Statistic | 0.78  (0.77, 0.79) |  | 0.73  (0.71, 0.76) | 0.82  (0.80, 0.84) | 0.75  (0.72, 0.78) | 0.78  (0.76, 0.80) |  | 0.83  (0.82, 0.85) |  | 0.83  (0.81, 0.85) | 0.86  (0.84, 0.88) | 0.78  (0.75, 0.81) | 0.81  (0.79, 0.83) |
| IDI*  (95% CI) | 0.07  (0.06, 0.08) |  | 0.04  (0.03, 0.05) | 0.10  (0.09, 0.12) | 0.07  (0.06, 0.09) | 0.10  (0.09, 0.12) |  | 0.01  (0.00, 0.01) |  | 0.00  (-0.00, 0.01) | 0.00  (-0.00, 0.01) | 0.01  (0.00, 0.01) | 0.01  (0.00, 0.01) |
| Continuous NRI*  (95% CI) | 0.68  (0.64, 0.74) |  | 0.58  (0.48, 0.69) | 0.83  (0.71, 0.92) | 0.59  (0.43, 0.69) | 0.75  (0.66, 0.87) |  | 0.52  (0.46, 0.57) |  | 0.46  (0.35, 0.56) | 0.67  (0.58, 0.78) | 0.32  (0.20, 0.44) | 0.42  (0.33, 0.52) |
| Event NRI*  (95% CI) | 0.32  (0.29, 0.37) |  | 0.32  (0.23, 0.42) | 0.40  (0.29, 0.50) | 0.29  (0.15, 0.37) | 0.37  (0.28, 0.47) |  | 0.11  (0.06, 0.15) |  | 0.07  (-0.03, 0.15) | 0.21  (0.12, 0.31) | -0.07  (-0.19, 0.04) | 0.04  (-0.05, 0.13) |
| Non-event NRI*  (95% CI) | 0.36  (0.34, 0.38) |  | 0.26  (0.22, 0.29) | 0.43  (0.41, 0.46) | 0.30  (0.25, 0.35) | 0.38  (0.34, 0.42) |  | 0.41  (0.39, 0.43) |  | 0.39  (0.35, 0.42) | 0.45  (0.43, 0.48) | 0.39  (0.35, 0.43) | 0.37  (0.34, 0.41) |

*IDI and NRI computed relative to Model A

** Predictors included in models are as follows: Model A: risk score only; Model B: HOMA-IR only; Model C: risk score and HOMA-IR; Model D risk score, HOMA-IR, and risk score by HOMA-IR interaction.

All models controlled for study site. HOMA-IR was standardized to facilitate comparability of OR’s with MetS severity; model fit/prediction statistics included scores on their original scale.

Statistically significant (p < 0.05) OR’s (different than 1) and IDI’s/NRI’s (different than 0) were shaded for ease of display
